# Radical Change in Zoonotic Abilities of Atypical BSE Prion Strains as Evidenced by Crossing of Sheep Species Barrier in Transgenic Mice

**DOI:** 10.3201/eid2606.181790

**Published:** 2020-06

**Authors:** Alba Marín-Moreno, Alvina Huor, Juan Carlos Espinosa, Jean Yves Douet, Patricia Aguilar-Calvo, Naima Aron, Juan Píquer, Sévérine Lugan, Patricia Lorenzo, Cecile Tillier, Hervé Cassard, Olivier Andreoletti, Juan María Torres

**Affiliations:** Centro de Investigación en Sanidad Animal, Madrid, Spain (A. Marín-Moreno, J.C. Espinosa, P. Aguilar-Calvo, J. Píquer, P. Lorenzo, J.M. Torres);; Interactions Hôte Agent Pathogène–École Nationale Vétérinaire de Toulouse, Toulouse, France (A. Huor, J.Y. Douet, N. Aron, S. Lugan, C. Tiller, H. Cassard, O. Andreoletti)

**Keywords:** transmissible spongiform encephalopathies, prion, atypical bovine spongiform encephalopathy, BSE, Val_129_-PrP, transmission barrier, Creutzfeldt-Jakob disease, CJD, zoonoses

## Abstract

Classical bovine spongiform encephalopathy (BSE) is the only zoonotic prion disease described to date. Although the zoonotic potential of atypical BSE prions have been partially studied, an extensive analysis is still needed. We conducted a systematic study by inoculating atypical BSE isolates from different countries in Europe into transgenic mice overexpressing human prion protein (PrP): TgMet_129_, TgMet/Val_129_, and TgVal_129_. L-type BSE showed a higher zoonotic potential in TgMet_129_ mice than classical BSE, whereas Val_129_-PrP variant was a strong molecular protector against L-type BSE prions, even in heterozygosis. H-type BSE could not be transmitted to any of the mice. We also adapted 1 H- and 1 L-type BSE isolate to sheep-PrP transgenic mice and inoculated them into human-PrP transgenic mice. Atypical BSE prions showed a modification in their zoonotic ability after adaptation to sheep-PrP producing agents able to infect TgMet_129_ and TgVal_129_, bearing features that make them indistinguishable of sporadic Creutzfeldt-Jakob disease prions.

Prion diseases, or transmissible spongiform encephalopathies (TSEs), are a group of rare and lethal neurodegenerative diseases that affect a great number of mammal species, including humans and animals belonging to the human food chain. The conversion of a host encoded cellular protein of unknown function (PrP^C^) into a disease-associated isoform (PrP^Sc^) is the molecular event underlying the development of TSEs. Such conformational change is driven by PrP^Sc^ itself because it recruits and transforms PrP^C^ molecules, acting as a template (*1*). The conformational change, associated with an increase of β-sheet content, also results in a change in the protein biochemical features (*2*). Although PrP^C^ is monomeric, protease-sensitive, and soluble in nonionic detergents, PrP^Sc^ has a high tendency to aggregate, is partially resistant to protease digestion, and is insoluble in nonionic detergents (*1*,*3*). 

Classical bovine spongiform encephalopathy (C-BSE) caused a major food safety crisis when consumption of contaminated meat was discovered in the late 1990s as the cause of a new prion disease affecting humans, which was called variant Creutzfeldt-Jakob disease (vCJD) (*4*). The first description of C-BSE was made in 1987 in affected cattle in the United Kingdom (*5*). In the years following, ≈200,000 cattle succumbed to the disease (*6*). To date, C-BSE is the only recognized zoonotic prion (*6*) and is responsible for >231 human deaths (*7*).

After the implementation of active surveillance in the European Union in 2001, several atypical BSE cases were identified in aged asymptomatic cattle during slaughterhouse testing. Two major phenotypes with pathology and epidemiology distinct from C-BSE were observed, bovine amyloidotic spongiform encephalopathy (or L-BSE) (*8*) and H-BSE (*9*). The biochemical properties of PrP^Sc^ isolated from these cases also differed from C-BSE in terms of the protease-resistant fragment size and ratio of glycoforms on Western blot (WB). It is unclear whether atypical BSE resulted from exposure to an acquired TSE or emerged spontaneously, a theory supported by the occurrence of atypical BSE being maintained at a similar rate in various countries independent of their C-BSE status.

The zoonotic potentials of atypical and C-BSE seemed to differ. One study performed in transgenic mice overexpressing the human Met_129_–normal brain prion protein (PrP) variant (Tg650) showed that L-BSE has a higher zoonotic potential than C-BSE because a 100% attack rate was observed in the intracranial challenge, whereas H-BSE was unsuccessfully transmitted (*10*). Other intermediate species belonging to the human food chain might play a role in a possible atypical BSE zoonosis. For example, C-BSE can naturally affect goats (*11*). Furthermore, C-BSE virulence in human-PrP transgenic mouse models is increased after passaging in ovine-PrP transgenic mice (*12*). The possible zoonotic potential of atypical BSE after its adaptation to the sheep sequence is not known. At least L-type BSE is efficiently transmitted in sheep (*13*). L-BSE transmission in ovine-PrP transgenic mouse models produced a prion similar to C-BSE in terms of incubation times, histopathologic features, and electrophoretic mobility, although the glycoprofile maintained a more equilibrated proportion between the 3 bands than C-BSE (*14*). Therefore, a deep assessment of the zoonotic potential of atypical BSE prions should include the evaluation of zoonotic potential after adaptation to the sheep-PrP sequence. 

Polymorphisms and mutations in the human prion protein gene affect survival and disease development in vCJD and other human TSEs (*15*). The most important genetic variant for disease outcome in humans is the polymorphic codon 129, which can codify methionine (Met_129_) or valine (Val_129_) and has been detected as Met_129_ homozygous in all vCJD-diagnosed cases, with the exception of 1 Met/Val_129_ heterozygous vCJD case (*16*,*17*).

The main aim of our study was to assess the zoonotic potential of the atypical BSE prions in transgenic mice that overexpress human-PrP covering the 3 possible 129 codon genotypes. We used a high number of isolates from different sites in Europe and a collection of human-PrP transgenic mouse lines. In addition, we adapted 1 H-BSE and 1 L-BSE isolate to the sheep-PrP sequence and sequentially inoculated them into human-PrP TgMet_129_ and TgVal_129_ mice to assess whether intermediate passage in sheep can modify prion strain features, including prion virulence in humans. We decided to use 2 different ovine-PrP transgenic mouse models to study the effect of the polymorphism Ala/Val_136_ of the sheep-PrP sequence.

## Materials and Methods

### Ethics Statement

We conducted animal experiments in accordance with the Code for Methods and Welfare Considerations in Behavioral Research with Animals (Directive 2010/63/EU) and made every effort to minimize suffering. Experiments developed in Centro de Investigación en Sanidad Animal–Instituto Nacional de Investigación y Tecnología Agraria y Alimentaria (Madrid, Spain) were evaluated by the Committee on the Ethics of Animal Experiments of the Instituto Nacional de Investigación y Tecnología Agraria y Alimentaria and approved by the General Directorate of the Madrid Community Government (permit nos. PROEX 263/15, PROEX 181/16, and PROEX 228/16). Experiments developed in Institut National de la Recherche Agronomique–École Nationale Vétérinaire de Toulouse (Toulouse, France) were approved by the Institut National de la Recherche Agronomique/École Nationale Vétérinaire de Toulouse Ethics Committee under the auspices of the Ministry of Education and Research of France (permit no. APAFIS-2017044210492380 v2).

### Prion Isolates

We used a collection of atypical BSE field isolates from different countries in Europe to ensure the consistency of the results (Appendix Table). We characterized all isolates to differentiate them from C-BSE (data not shown). For comparison, we included C-BSE isolates and other prion isolates in the study. For inoculation, we prepared all isolates from brain tissues as 10% (wt/vol) homogenates in 5% glucose. Second passages were performed by inoculating mice with 10% (wt/vol) homogenates in 5% glucose of brains selected from first passage.

### Mouse Transmission Studies

The atypical BSE isolates were inoculated in 3 different mouse models for human-PrP: HuPrP-Tg340-Met_129_ (TgMet_129_) line expressing human Met_129_-PrP variant (*12*), HuPrP-Tg361-Val_129_ (TgVal_129_) line expressing human Val_129_-PrP variant (*18*), and HuPrP-Tg351-Met/Val_129_ (TgMet/Val_129_) line obtained by mating TgMet_129_ and TgVal_129_ mice (*18*). All of these transgenic lines show similar brain PrP^C^ levels of expression (≈4-fold the level of expression in human brain) on a mouse-PrP null background. For the adaptation to the sheep-PrP sequence, we used 2 different overexpressing sheep-PrP mouse models to include the 2 variants of the Ala/Val_136_ Arg_154_ Gln_171_ haplotype. The OvPrP-Tg338 (TgVRQ) harbors the VRQ allele and expresses 8–10-fold the level of PrP^C^ expression in sheep brain (*19*). The OvPrP-TgShXI (TgARQ) harbors the ARQ allele and expresses 3–4-fold the level of PrP^C^ expression in sheep brain (*20*). We performed subsequent bioassays for the detection of low-level propagation of atypical BSE prions in BoPrP-Tg110 mice (TgBo) (*21*).

We individually anesthetized 6–7-week-old mice with isoflurane and inoculated them with 2 mg equivalent of brain homogenate in the right parietal lobe by using a 25-gauge disposable hypodermic needle. We observed mice daily and assessed their neurologic status twice a week. When progression of a TSE disease was evident or at the established experimental endpoint (700 days postinoculation), we euthanized the animals for ethical reasons, then performed necropsy and removed the brain. We fixed part of the brain by using immersion in neutral-buffered 10% formalin (4% 2-formaldehyde) and used it for histopathology; we froze the rest of the tissue at −20°C and used it for determining the presence of proteinase K–resistant PrP^Sc^ (PrP^res^) by WB. We calculated survival times as mean + SD days postinoculation for all the mice that scored positive for PrP^res^. We defined the attack rate as the proportion of mice that scored positive for PrP^res^ divided by the number of inoculated mice. We used brain homogenates from PrP^res^-positive mice for further passaging. When all mice were scored negative for PrP^res^ on primary passage, we pooled all PrP^res^-negative brains and used them for second passage.

### Histologic Examination and Paraffin-Embedded Tissue Blotting

We performed all procedures comprising the histopathologic analysis of mouse brains as previously described (*22*). We fixed mouse brain samples in neutral-buffered 10% formalin (4% 2-formaldehyde) and embedded them in paraffin. After deparaffinization, we stained 4-µm-thick tissue slices with hematoxylin and eosin. We established brain lesion profiles according to published methods (*23*). We conducted paraffin-embedded tissue (PET) blots as previously described (*24*) by using the Sha31 monoclonal antibody (mAb) (*25*).

### Western Blotting

We homogenized frozen brain tissues (175 + 20 mg) in 5% glucose in distilled water in grinding tubes (Bio-Rad, https://www.bio-rad.com) adjusted to 10% (wt/vol) by using a TeSeE Precess 48TM homogenizer (Bio-Rad). We determined the presence of PrP^res^ in transgenic mouse brains by using WB, using the reagents of the ELISA commercial test TeSeE (Bio-Rad). We prepared brain homogenates (10–100 µL of a 10% [wt/vol]) as previously described (*18*) and loaded samples into 12% Bis-Tris Gel (Criterion XT; Bio-Rad). We transferred proteins electrophoretically onto polyvinylidene fluoride membranes (Millipore, https://www.sigmaaldrich.com) and blocked overnight with 2% bovine serum albumin blocking buffer. We incubated membranes with Sha31 (*25*) mAb at a concentration of 1 µg/mL. Sha31 recognizes the 145-WEDRYYRE-152 epitope of the human-PrP^C^ sequence, which is conserved in sheep and bovine sequences. We detected immunocomplexes by incubating the membranes for 1 h with horseradish peroxidase conjugated antimouse IgG (GE Healthcare Amersham Biosciences, https://www.gelifesciences.com). We developed immunoblots with enhanced chemiluminescence in ECL Select (GE Healthcare Amersham Biosciences) and captured images by using the ChemiDoc WRS+ System and processed them by using Image Lab 5.2.1 software (both Bio-Rad).

## Results

### Atypical BSE Transmission to Human-PrP Transgenic Mouse Models

We transmitted a panel of atypical L-type and H-type BSE (2 serial passages) into 3 transgenic mouse lines. These mouse lines were homozygous for Met (TgMet_129_) or Val (TgVal_129_) at codon 129 of human-PrP or were their F1 cross resulting in heterozygous mice (TgMet/Val_129_). The PrP^C^ level in the brain of all 3 transgenic mice lines has been shown to be approximately 4-fold higher than in human brain tissue (*26*). In parallel, we inoculated C-BSE control isolates (Table 1).

**Table 1:** Transmission of H- and L-type BSE isolates to TgMet129, TgMet/Val129, and TgVal129 mice in a study of atypical BSE transmission using isolates from different countries in Europe and transgenic mouse models overexpressing human normal brain prion protein*

Isolate	Mean survival period, dpi + SD (n/n_0_) (reference)†							
	TgMet_129_			TgMet/Val_129_			TgVal_129_	
	P1	P2		P1	P2		P1	P2
C-BSE_0_	739 (1/6) (*12**,**18*)	633 + 32 (6/6) (*18*)		>700 (0/6) (*18*)	>700 (0/6) (*18*)		>700 (0/6) (*18*)	>700 (0/6) (*18*)
C-BSE_2_	491–707 (0/9) (*12**,**18*)	572 + 37 (3/4) (*12**,**18*)		>700 (0/5) (*18*)	ND		>700 (0/6) (*18*)	>700 (0/6) (*18*)
C-BSE_3_	758–801 (2/6)	615 + 95 (6/6)		ND	ND		>700 (0/6)	>700 (0/6)
BSE L_1_	607 + 13 (7/7)	487 + 116 (4/4)		>700 (0/12)	ND		>700 (0/14)	>700 (0/4)
BSE L_1_→TgMet_129_	487 + 116 (4/4)	ND		ND	ND		>700 (0/4)	ND
BSE L_2_	629 + 35 (7/7)	508 + 97 (5/5)		>700 (0/6)	ND		>700 (0/6)	>700 (0/6)
BSE L_2_→TgMet_129_	508 + 97 (5/5)	ND		>700 (0/7)	ND		>700 (0/6)	>700 (0/6)
BSE L_3_	541 + 70 (7/7)	ND		>700 (0/11)	ND		>700 (0/11)	ND
BSE H_1_	>700 (0/19)	>700 (0/6)		>700 (0/14)	ND		>700 (0/13)	>700 (0/6)
BSE H_2_	>700 (0/12)	>700 (0/6)		>700 (0/12)	ND		>700 (0/12)	ND
BSE H_3_	>700 (0/14)	>700 (0/12)		>700 (0/12)	ND		>700 (0/12)	ND

*BSE, bovine spongiform encephalopathy; C-BSE, classical bovine spongiform encephalopathy; dpi, days postinoculation; ND, not detected; n/n_0_, diseased proteinase K–resistant prion protein–positive/inoculated animals; P1, first passage; P2, second passage. †Survival time is indicated as mean dpi + SD for all mice that scored positive for proteinase K–resistant prion protein.

As we reported on a previous study (*18*), only TgMet_129_ mice get infected with C-BSE isolates. However, TgMet/Val_129_ and TgVal_129_ showed no transmission or accumulation of PrP^res^ (Table 1).

**Figure 1:**
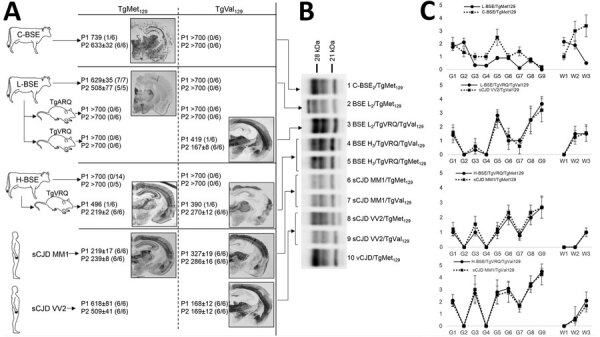
Atypical BSE transmission into human-PrP transgenic mice before and after adaptation to sheep PrP sequence in a study of atypical BSE transmission using isolates from different countries in Europe and transgenic mouse models overexpressing human normal brain prion protein. A) Transmission data including mean survival time + SD as well as attack rates (diseased PrP^res^ positive/inoculated animals) and PET blot images for all atypical BSE transmission into the human-PrP transgenic mouse models. L-BSE/TgMet_129_ showed fine staining, and deposits were restricted to the several thalamus nuclei. C-BSE/TgMet129 showed granular deposits. L-BSE/TgVRQ/TgVal_129_ and TgVal_129_ PET blotting showed strong deposition in a particular area of the isocortex, thalamus, and midbrain, and mild deposition in the fiber tracts. H-BSE/TgVRQ and sCJD MM1 PET blotting images showed strong deposition in the isocortex area, hippocampus, thalamus, and midbrain in TgMet_129_ and strong deposition in the isocortex area, thalamus, and midbrain in TgVal_129_. B) Brain PrP^res^ profile in TgMet_129_ and TgVal_129_ mice inoculated with atypical BSE prions before or after adaptation to the sheep-PrP sequence immunoblotted with the Sha31 mAb. Human vCJD and different sCJD prion strains inoculated in the same TgMet_129_ and TgVal_129_ mouse models are also included for comparison purposes. L-BSE/TgVRQ/TgVal_129_ (lane 3) is very similar to sCJD VV2/TgVal_129_ (lane 9). By contrast, H-BSE/TgVRQ/TgMet_129_ (lane 5) and sCJD MM1/TgMet_129_ (lane 6) are undistinguishable, as also observed with H-BSE/TgVRQ/TgVal_129_ (lane 4) and sCJD MM1/TgVal_129_ (lane 7). All PrP^res^ profiles are different from those of vCJD/TgMet_129_ (lanes 1 and 10) and L-BSE/TgMet_129_ (lane 2). All inoculated animals were analyzed, and individual variations in the PrP^res^ profile among them were not found. C) Vacuolar lesion profile in brains from human-PrP transgenic mice inoculated with C-BSE or the atypical BSE isolates before and after adaptation to the sheep-PrP sequence. Lesion scoring was conducted for 9 areas of gray matter (G) and 3 areas of white matter (W) in mouse brains: G1, dorsal medulla; G2, cerebellar cortex; G3, superior colliculus; G4, hypothalamus; G5, medial thalamus; G6, hippocampus; G7, septum; G8, medial cerebral cortex at the level of the thalamus; G9, medial cerebral cortex at the level of the septum (G9); W1, cerebellum; W2, mesencephalic tegmentum; W3, pyramidal tract. BSE, bovine spongiform encephalopathy; C-BSE, classical bovine spongiform encephalopathy; mAb, monoclonal antibody; PET, paraffin embedded tissue; PrP, prion protein; PrPres, proteinase K–resistant prion protein; sCJD, sporadic Creutzfeldt-Jakob disease; vCJD, variant Creutzfeldt-Jakob disease.

L-BSE was efficiently transmitted to TgMet_129_ (Table 1; Figure 1, panel A). The attack rate was 100% for first passage, and incubation time was not reduced on subsequent passaging. In TgMet_129_, the L-BSE PrP^res^ pattern in WB differed from the C-BSE PrP^res^ pattern both in terms of apparent molecular weight and glycoprofile distribution, marked by an evident lower proportion of the diglycosylated band (Figure 1, panel B; Appendix Figure 1). We examined the regional distribution and intensity of PrP^res^ deposition in the brain by using PET blotting. Brains of TgMet_129_ inoculated with L-BSE showed finer staining than the granular PrP deposits typical of C-BSE (Figure 1; Appendix Figure 1). Deposits were mostly restricted to the habenular, geniculate, and dorsal nuclei of the thalamus. Lesion profiles also reflect differences between both strains (Figure 1, panel C). We detected no clinical signs, PrP^res^ accumulation by WB (Table 1), or PrP^Sc^ deposition by PET blotting (data not shown) in the brains of TgVal_129_ or TgMet/Val_129_ L-BSE inoculated mice. These brains were collected and inoculated in TgBo animals; no transmission was observed (data not shown). This finding suggests the absence of subclinical infection in TgVal_129_ and TgMet/Val_129_ mice. We inoculated L-BSE passaged in TgMet_129_ into TgVal_129_ and TgMet/Val_129 _mice and detected no PrP^res^ accumulation (Table 1). After H-BSE inoculation, we detected no clinical signs, PrP^res^ accumulation by WB (Table 1), or PrP^Sc^ deposits by PET blotting (data not shown) in any of the 3 human transgenic mouse models.

### Atypical BSE Transmission into Ovine-PrP Transgenic Mouse Models

We transmitted 1 L-BSE isolate and 1 H-BSE isolate (2 iterative passages) to VRQ and ARQ ovine-PrP transgenic mice. L-BSE transmitted in both TgVRQ and TgARQ caused 100% attack rates and short incubation times in the second passage (Table 2).

**Table 2:** Intracerebral inoculation of TgBo, TgVRQ, and TgARQ mice with atypical BSE isolates to promote adaptation to the sheep-PrP sequence in a study of atypical BSE transmission using isolates from different countries in Europe and transgenic mouse models overexpressing human normal brain prion protein*

Isolate	Mean survival time, d + SD (n/n_0_)†							
	TgBo			TgVRQ			TgARQ	
	P1	P2		P1	P2		P1	P2
BSE L_2_	263 + 31 (6/6)	208 + 21 (6/6)		438 + 20 (6/6)	168 + 22 (6/6)		386‡, 404 (2/6)	155 + 8 (6/6)
BSE H_3_	382 + 74 (6/6)	262 + 3 (6/6)		801 (1/6)	408 + 44 (6/6)		>700 (0/6)	>700 (0/6)

*BSE, bovine spongiform encephalopathy; dpi, days post-inoculation; n/n_0_, diseased proteinase K–resistant prion protein–positive/inoculated animals; P1, first passage; P2, second passage. †Survival time is indicated as mean dpi + SD for all mice that scored positive for proteinase K–resistant prion protein. ‡Found dead animals without clinical signs and positive for proteinase K–resistant disease-associated isoform.

**Figure 2:**
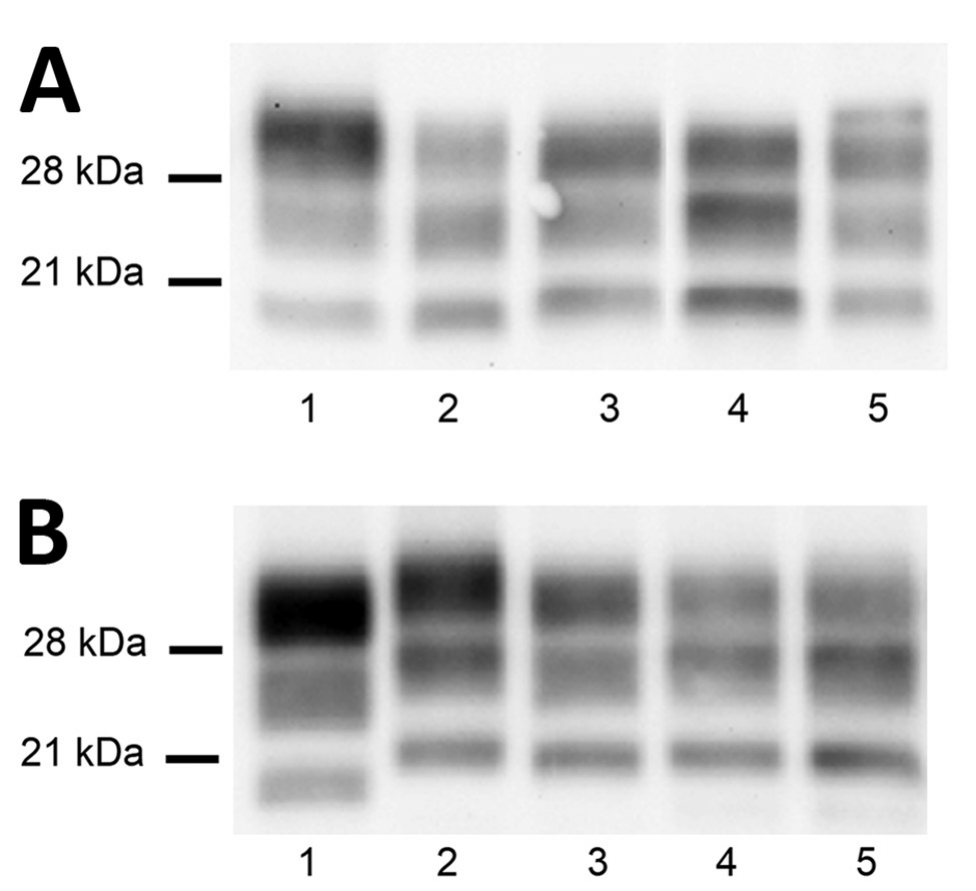
Atypical BSE transmission into sheep PrP transgenic mice in a study of atypical BSE transmission using isolates from different countries in Europe and transgenic mouse models overexpressing human normal brain prion protein. A) Brain PrP^res^ profile of L-BSE prions (lane 2) changed once passaged into TgVRQ (lane 3) and TgARQ (lane 5). L-BSE propagation into TgVRQ and TgARQ produced a PrP^res^ profile with a molecular weight slightly higher than C-BSE (lane 1). L-BSE/TgVRQ transmission into TgVal_129_ mice rendered a PrP^res^ similar to type 2 sCJD profile when transmitted in the same animal model (lane 4). All inoculated animals were analyzed and individual variations in the PrP^res^ profile among them were not found. Lane 1, C-BSE_2_; lane 2, BSE L_2_; lane 3, BSE L_2_/TgVRQ; lane 4, BSE L_2_/TgVRQ/TgVal129; lane 5, BSE L_2_/TgARQ. B) Brain PrP^res^ profile of H-BSE prions (lane 2) changed once passaged into TgVRQ (lane 3) and produced a 21 kDa PrP^res^ profile very different from that of C-BSE (lane 1). H-BSE/TgVRQ transmission into TgMet_129_ (lane 4) and TgVal_129_ (lane 5) mice rendered a PrP^res^ similar to type 1 sCJD profile when transmitted in the same animal models. All inoculated animals were analyzed and individual variations in the PrP^res^ profile among them were not found. Lane 1, C-BSE_2_; lane 2, BSE H_3_; lane 3, BSE H_3_/TgVRQ; lane 4, BSE H_3_/TgVRQ/TgMet129; lane 5, BSE H_3_/TgVRQ/TgVal129. BSE, bovine spongiform encephalopathy; C-BSE, classical bovine spongiform encephatlopathy; PrP, prion protein; PrPres, proteinase K–resistant prion protein; sCJD, sporadic Creutzfeldt-Jakob disease.

**Figure 3:**
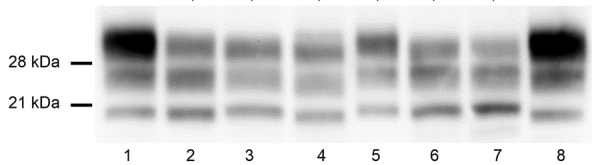
Bovine-PrP transgenic mice challenged with atypical BSEs transmitted into human-PrP transgenic mice before and after adaptation to sheep-PrP sequence in a study of atypical BSE transmission using isolates from different countries in Europe and transgenic mouse models overexpressing human normal brain prion protein. Brain PrP^res^ in TgBo mice inoculated with different atypical BSE either before or after passage into the different transgenic lines. L-BSE biochemical profile (lane 2) changed once passaged into TgVRQ (lane 3) and TgARQ (lane 4). L-BSE/TgVRQ produced a PrP^res^ profile resembling the one of C-BSE (lanes 1 and 8). L-BSE propagation into TgARQ produced a 21kDa PrP^res^ profile. H-BSE (lane 5) can still infect back TgBo line once passaged into TgVRQ and adapted to the human PrP sequence (lanes 6 and 7) and produced a 21 kDa PrP^res^ profile. All inoculated animals were analyzed and individual variations in the PrP^res^ profile among them were not found. Lane 1, C-BSE_2_; lane 2, BSE L_2_; lane 3, BSE L_2_/TgVRQ/TgBo; lane 4, BSE L_2_/TgARQ/TgBo; lane 5, BSE H_3_; lane 6, BSE H_3_/TgVRQ/TgMet129/TgBo; lane 7, BSE H_3_/TgVRQ/TgVal129/TgBo; lane 8, C-BSE_2_. BSE, bovine spongiform encephalopathy; C-BSE, classical bovine spongiform encephatlopathy; PrP, prion protein; PrPres, proteinase K–resistant prion protein.

L-BSE propagation in all TgVRQ and TgARQ infected mice showed a WB PrP^res^ profile with similarities to the one observed after passage of C-BSE in the same model in terms of low molecular mass migration but also a glycoform ratio where the diglycosylated and monoglycosylated bands contain a more equal signal proportion than C-BSE were detected with mAb Sha31, which is more similar to L-BSE or classical scrapie (Figure 2, panel A). These features have previously been reported in ARQ/ARQ and VRQ/VRQ sheep inoculated with L-BSE (*13*). We inoculated brains collected from the second passage in TgBo, causing a clinical disease with similar incubation periods as original L-BSE (Table 3). Both WB and PET blotting patterns (Appendix Figure 2), as well as WB patterns once transmitted back into TgBo (Figure 3), support the view that passage in ovine sequences did not irreversibly alter L-BSE strain properties.

**Table 3:** Intracerebral inoculation of TgBo with sCJD, L-BSE, and H-BSE isolates after their adaptation (P2) in various hosts in a study of atypical BSE transmission using isolates from different countries in Europe and transgenic mouse models overexpressing human normal brain prion protein*

Isolate	Mean survival time, days + SD (n/n_0_)†	
	P1	P2
BSE L_2_	263 + 31 (6/6)	204 + 4 (6/6)
BSE L_2_→TgVRQ (P2)	221 + 3 (6/6)	212 + 3 (6/6)
BSE L_2_→TgARQ (P2)	240 + 18 (6/6)	215 + 5 (6/6)
BSE L_2_→TgVRQ (P2)→TgVal_129_ (P2)	>650 (0/6)	ND
BSE H_3_	382 + 74 (6/6)	262 + 3 (6/6)
BSE H_3_→TgVRQ (P2)	>650 (0/6)	>650 (0/6)
BSE H_3_→TgVRQ (P2)→TgMet_129_ (P2)	671, 699,‡ 759‡ (3/6)	631 + 34 (5/6)
BSE H_3_→TgVRQ (P2)→TgVal_129_ (P2)	627,‡ 689‡ (2/6)	703‡ (1/6)
sCJD MM1→TgMet_129_ (P2)	750 + 18 (3/6)	ND
sCJD MM1→TgVal_129_ (P2)	737, 763,‡ 833‡ (3/4)	ND
sCJD VV2→TgVal_129_ (P2)	833‡ (1/6)	ND
vCJD→TgMet_129_ (P2)	249 + 2 (6/6)	236 + 5 (6/6)

*BSE, bovine spongiform encephalopathy; dpi, days post-inoculation; n/n0, diseased proteinase K–resistant prion protein–positive/inoculated animals; P1, first passage; P2, second passage, sCJD, sporadic Creutzfeldt-Jakob disease; vCJD, variant Creutzfeldt-Jakob disease. †Survival time is indicated as mean dpi + SD for all mice that scored positive for proteinase K–resistant prion protein. ‡Found dead animals without clinical signs and positive for proteinase K–resistant disease-associated isoform.

TgARQ mice inoculated with H-BSE isolate did not show any clinical sign or accumulation of PrP^res^ (Table 2). In TgVRQ, first passage produced disease in only 1 out of 6 animals, with long incubation times. On second passage, 100% attack rates were achieved, and incubation times were reduced (Table 2). This finding suggests a substantial transmission barrier for the H-BSE prion agent in this animal model. The WB PrP^res^ profile was characterized by 21 kDa profile (Figure 2, panel B). The prion that propagated in TgVRQ mice inoculated with H-BSE could not be transmitted (2 serial passages) in TgBo mice. These results suggest that, upon adaptation to the VRQ ovine-PrP sequence, the H-BSE strain properties were irreversibly altered (Table 3).

We thus analyzed the neuropathologic phenotypes of the atypical BSE agents transmitted into ovine-PrP transgenic mice by PrP^res^ PET blotting (Appendix Figure 2). Regarding L-BSE, the results support those obtained by WB because TgVRQ ovine passaged L-BSE displays a similar PrP deposition as ovine passaged C-BSE. PrP^res^ deposition predominantly involved several nuclei of the thalamus and regions like the septum and external cortex of the inferior colliculus; we obtained similar results for TgARQ passaged L-BSE (Appendix Figure 2), as previously described (*14*). Concerning TgVRQ adapted H-BSE prions, the deposition pattern is also strikingly different to that displayed by C-BSE or L-BSE (Appendix Figure 2).

### Atypical BSE Transmission into Human-PrP Transgenic Mouse Models after Adaptation to the Sheep-PrP Sequence

We inoculated prions obtained after 2 passages of L-BSE and H-BSE isolates in ovine PrP expressing mice into TgMet_129_ and TgVal_129_. TgMet_129_ mice inoculated with L-BSE passaged into TgVRQ showed neither clinical signs nor PrP^res^ accumulation in their brain (Figure 1, panels A, B). By contrast, TgVal_129_ inoculated with the same isolates had disease characterized by 100% attack rates and short incubation times in the second passage (Figure 1, panel A). The obtained PrP^res^ resembles a sporadic Creutzfeldt-Jakob disease (sCJD) Val/Val_129_ type 2 (sCJD VV2) profile (Figure 1, panel B). PET blotting showed a deposition pattern similar to that of sCJD VV2 in the same mice (Figure 1, panel A). Lesion profiles of both strains also were coincident (Figure 1, panel C). Furthermore, the prion generated in the TgVal_129_ transgenic mouse line is not able to infect back in TgBo (Table 3). By contrast, ARQ-adapted L-BSE loses its ability to infect human-PrP TgMet_129_ transgenic mouse lines (Figure 1, panel A).

VRQ-adapted H-BSE can now infect both human-PrP TgMet_129_ and TgVal_129_ mouse lines, showing 100% attack rates and short incubation times in the second passage (Figure 1, panel A). The obtained PrP^res^ in both cases is similar to that of sCJD Met/Met_129_ type 1 (sCJD MM1) (Figure 1, panel B). PET blotting showed similarities with the deposition patterns typical of sCJD MM1 in TgMet_129_ and TgVal_129_ (Figure 1, panel A). Lesion profiles of both strains also were coincident (Figure 1, panel C). Both generated prions can infect back in TgBo (Table 3; Figure 3).

VRQ-adapted L-BSE reduces its zoonotic potential toward human-PrP TgMet_129_ mice, as shown by a total abolishment of prion replication in TgMet_129_ (Figure 1, panel A). Strikingly, intermediate passage of L-BSE into TgVRQ increased its zoonotic potential toward TgVal_129_ mice (Figure 1, panel A).

## Discussion

The zoonotic potential of atypical BSE prions has been partially studied by using both PrP-overexpressing animals and gene-targeted mice (*10*,*27*–*29*). All evidence converges to indicate a higher capacity of L-BSE than of C-BSE to transmit in human-PrP–expressing hosts and a high transmission barrier for H-BSE. Absence of a transmission barrier for L-BSE in TgMet_129_ has already been reported (*10*). Our study (using other transgenic mice with a different PrP^C^ expression level) expands the investigation to other genotypes. Lack of prion propagation in TgMet/Val_129_ and TgVal_129_ indicates that Val_129_ variant is a strong molecular protector against L-BSE zoonotic transmission even in heterozygosis, as previously reported for C-BSE and related C-BSE prions (*18*). Finally, H-BSE clearly has a robust transmission barrier with respect to the human-PrP sequence, independent of the codon 129 polymorphism.

Once adapted to TgMet_129_, L-BSE did not propagate in TgMet/Val_129_ and TgVal_129_, which precludes secondary infections between persons. In contrast, C-BSE propagated in Val_129_ genotypes once adapted to the Met_129_ human-PrP sequence, even when Val_129_ also protected against primary infection (*18*). However, Met/Val_129_ genotypes might be naturally affected by vCJD because a definite vCJD case of a subject heterozygous for codon 129 has already been reported (*16*). Moreover, evidence of potential prion propagation in Val_129_-homozygous persons has been indicated in examinations of tonsils and appendices (*30*–*32*). These observations are in contrast with our finding of a lack of transmission of C-BSE in Val_129_ genotypes. However, only 1 vCJD case has been reported in a Met/Val-heterozygous person, which might mean that the event is very rare. Moreover, whether the PrP^res^ positivity detected in lymphoid tissues (tonsils and appendix) of Val_129_-homozygous persons would eventually extend to their central nervous system is still unknown. As a consequence, the risk for L-BSE secondary transmission once adapted to human-PrP sequence should be assessed carefully.

A complete assessment of the zoonotic potential of atypical BSE prions should include the evaluation of zoonotic potential after adaptation to the sheep-PrP sequence given that C-BSE virulence toward human-PrP transgenic mouse models increased after passage in ovine-PrP transgenic mice. Thus, we propagated 1 H-BSE and 1 L-BSE isolate into ovine-PrP transgenic mice. L-BSE has already been transmitted into sheep (*13*), whereas no H-BSE propagation into this host has been reported. Thus, we decided to perform the adaptation to the sheep sequence by using sheep-PrP transgenic mice, although overexpression of PrP^C^ reportedly could render changes on prion strains features (*33*). The absence of divergent PrP^res^ profiles among the animals and the uniformity of the incubation times after 2 passages into sheep-PrP transgenic mice argue against the occurrence of mutation events attributable to PrP^C^ overexpression in our study. In addition, previously reported strain features of L-BSE propagated in sheep (*13*) were similar to those reported in this study using sheep-PrP transgenic mice, which also validates use of these animal models. Our results suggest a moderate but surmountable transmission barrier for L-BSE in the 2 analyzed sheep genotypes, whereas for H-BSE a high transmission barrier exists when transmitted into an ARQ sheep sequence. The polymorphism Ala/Val_136_ present in the sheep-PrP sequence seems to be responsible for the different behavior of the obtained prion agents. Once proved that these isolates could be propagated on sheep-PrP sequence, determining whether they can be differentiated from classical scrapie and C-BSE will be important. 

Our results and those provided by other studies indicate that L-BSE adapted to a VRQ sheep sequence resemble C-BSE in its molecular features (*14*). Moreover, L-BSE adapted to ARQ sheep sequence and H-BSE adapted to VRQ sheep sequence generate prions similar to classical scrapie, at least in terms of PrP^res^ glycoprofile. Therefore, in the supposed case of atypical BSE transmission to sheep, early differentiation of both atypical BSE agents from other sheep prions like classical scrapie would be difficult. Nevertheless, the combination of the low incidence of atypical BSE (because of its supposed sporadic nature) and the continued prohibition of meat and bone meal recycling ameliorates the risk for transmission to sheep.

The transmission of atypical BSEs into sheep resulted in the emergence of prions similar to types 1 and 2 sCJD in terms of mean survival times, attack rates, PrP^res^ profile, and PrP^res^ deposition pattern in the brain of human-PrP transgenic mice. The similarities between the sheep-adapted atypical BSE prions propagated into our human-PrP transgenic mouse lines and sCJD prions could suggest a link between them. The well-established dogma that sCJD is a spontaneous disorder unrelated to animal prion disease has been questioned in a previous study given the resemblance of scrapie prions transmitted into human transgenic mouse models to sCJD strains (*26*); however, the data from that study do not unequivocally establish a causative link between exposure to sheep scrapie and the subsequent appearance of sCJD in humans, and the same could apply to our findings. An alternative explanation that cannot be ruled out is that, although being different strains, only a limited number of phenotypes could be generated for the human-PrP, indicating phenotypic convergence. Updates to old epidemiologic research is needed to reconsider all these results involving a possible infectious origin of sCJD. In any case, continuing the characterization of this newly emerged prion strain would be useful to finally discarding or refuting a link with sCJD prions.

Extrapolation of results from prion transmission studies based on transgenic mice should be done with caution, especially when human susceptibility to prions is analyzed. However, our results clearly indicate that atypical BSE adaptation to an ovine-PrP sequence could modify the prion agent to potentially infect humans, showing strain features indistinguishable from those of classic sCJD prions, even though they might or might not be different agents. The supposed sporadic nature of atypical BSE makes its transmission to sheep and later to humans unlikely. However, the expanding range of TSE agents displaying the capacity to transmit in human-PrP–expressing hosts warrants the continuation of the ban on meat and bone meal recycling and underscores the ongoing need for active surveillance.

Appendix Additional information about radical change in zoonotic abilities of atypical BSE prion strains as evidenced by crossing of sheep species barrier in transgenic mice.
