# Supplementary material for: Radical Change in Zoonotic Abilities of Atypical BSE Prion Strains as Evidenced by Crossing of Sheep Species Barrier in Transgenic Mice
Source: Emerg Infect Dis. 2020 Jun;26(6):1130–9. doi: 10.3201/eid2606.181790 (PMC7258450; doi:10.3201/eid2606.181790)
Supplement: Appendix — Additional information about radical change in zoonotic abilities of atypical BSE prion strains as evidenced by crossing of sheep species barrier in transgenic mice. [file 18-1790-Techapp-s1.pdf]

Article DOI: https://doi.org/10.3201/eid2606.181790

# Radical Change in Zoonotic Abilities of Atypical BSE Prion Strains as Evidenced by Crossing of Sheep Species Barrier in Transgenic Mice

## Appendix

**Appendix Table.** Description of the different prion isolates used in this study

| Isolate | Description and references | Supplier |
|---|---|---|
| C-$BSE_0$ | BSE naturally infected cow (*1,2*) | INRA* |
| C-$BSE_2$ | BSE naturally infected cow (*1,2*) | VLA† |
| C-$BSE_3$ | BSE naturally infected cow | ENVT‡ |
| BSE $L_1$ | Brainstem of naturally affected cow diagnosed as L-BSE | NVRI§ |
| BSE $L_2$ | Brainstems of naturally affected cows diagnosed as L-BSE (*3*) | AFSSA¶ |
| BSE $L_3$ | BSE L2 isolate amplified in BoPrP-Tg110 mice (*3*) | AFSSA¶ |
| BSE $H_1$ | Brainstem of naturally affected cow diagnosed as H-BSE (*3*) | NVRI§ |
| BSE $H_2$ | Brainstem of naturally affected cow diagnosed as H-BSE | INIAV# |
| BSE $H_3$ | Brainstem of naturally affected cow diagnosed as H-BSE (*4*) | AFSSA¶ |

*French National Institute for Agricultural Research (INRA), Nouzilly, France.
†Veterinary Laboratory Agency (VLA), New Haw. Addlestone, Surrey, United Kingdom.
‡École Nationale Vétrinaire de Toulouse (ENVT), Toulouse, France.
§Polish National Veterinary Research Institute (NVRI), Pulawy, Poland.
¶National TSE Reference Laboratory, Agence Française de Sécurité Sanitaire des Aliments(AFSSA), Lyon, France.
#Instituto Nacional de Investigação Agrária e Veterinária (INIAV), Portugal.

### References


1. Padilla D, Béringue V, Espinosa JC, Andreoletti O, Jaumain E, Reine F, et al. Sheep and goat BSE propagate more efficiently than cattle BSE in human PrP transgenic mice. PLoS Pathog. 2011;7:e1001319. PubMed https://doi.org/10.1371/journal.ppat.1001319

2. Fernández-Borges N, Espinosa JC, Marín-Moreno A, Aguilar-Calvo P, Asante EA, Kitamoto T, et al. Val129-PrP variant is a strong molecular protector against BSE zoonotic transmission but fails to prevent human-to-human vCJD transmission. Emerg Infect Dis. 2017;23:1522–30. PubMed

3. Torres JM, Castilla J, Pintado B, Gutiérrez-Adan A, Andréoletti O, Aguilar-Calvo P, et al. Spontaneous generation of infectious prion disease in transgenic mice. Emerg Infect Dis. 2013;19:1938–47. PubMed https://doi.org/10.3201/eid1912.130106

4. Torres JM, Andréoletti O, Lacroux C, Prieto I, Lorenzo P, Larska M, et al. Classical bovine spongiform encephalopathy by transmission of H-type prion in homologous prion protein context. Emerg Infect Dis. 2011;17:1636–44. PubMed https://doi.org/10.3201/eid1709.101403

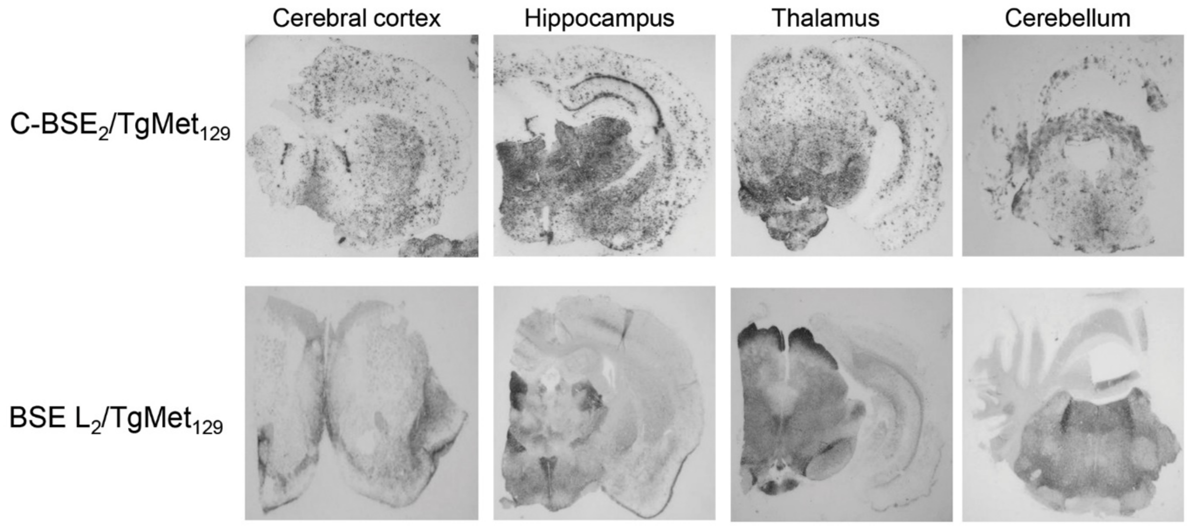


**Appendix Figure 1.** Analysis of $PrP^{res}$ distribution in the brains of L-BSE $TgMet_{129}$ inoculated mice by PET blotting. One representative $TgMet_{129}$ animal inoculated with L-BSE was selected for the figure. All L-BSE isolates propagated in $TgMet_{129}$ mice showed finer staining compared to the characteristic granular PrP deposits of $TgMet_{129}$ animals inoculated with C-BSE. L-BSE $PrP^{res}$ deposits were mainly found in the habenular, geniculate, and dorsal nuclei of the thalamus. Panels show cerebral cortex, hippocampus, thalamus and cerebellum sections.

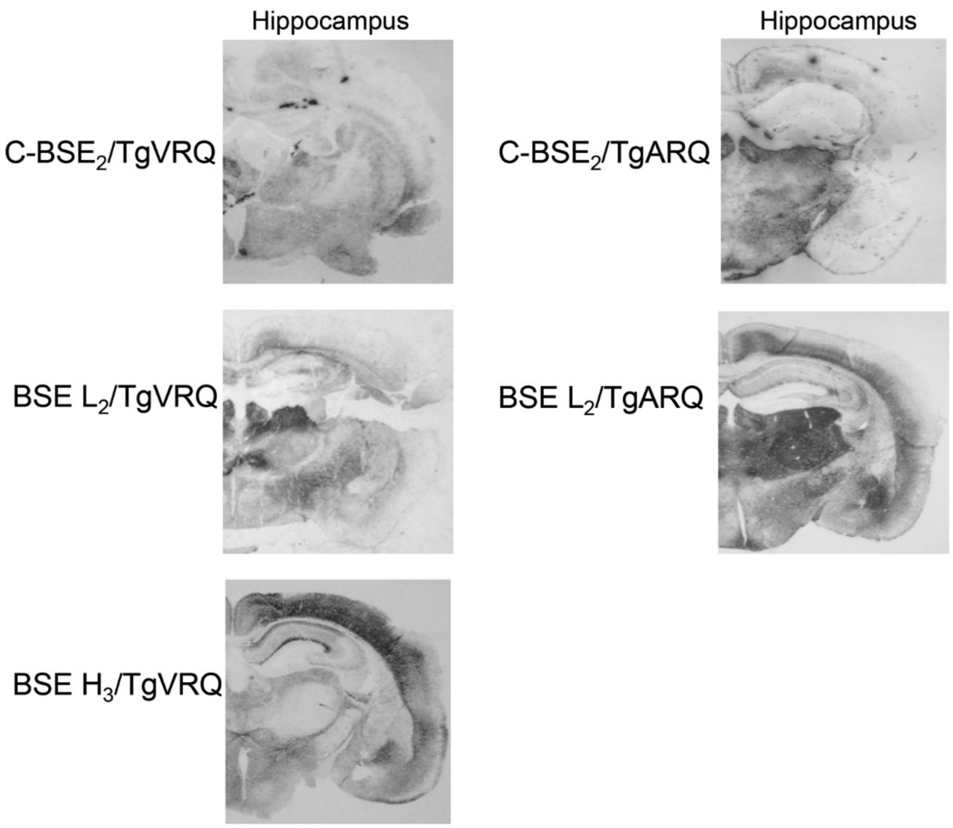


**Appendix Figure 2.** Analysis of $PrP^{res}$ distribution in the brains of TgVRQ and TgARQ inoculated mice by PET blotting. Representative animals inoculated with the different C-BSE and atypical BSE isolates were selected for the figure. L-BSE/TgVRQ and L-BSE/TgARQ showed similarities to C-BSE/TgVRQ in terms of the $PrP^{res}$ deposition pattern with deposits in thalamus nuclei, septum and external cortex of the inferior colliculus. By contrast, H-BSE/TgVRQ is remarkably different mainly characterized by an increase in the deposition in the isocortex area of the cerebral cortex. Panels show hippocampus sections.
